# Exploring the diversity of microbes and natural products from fungus-growing termite tripartite symbiosis

**DOI:** 10.1016/j.engmic.2023.100124

**Published:** 2023-11-04

**Authors:** Muhammad Shoaib, Ruining Bai, Shuai Li, Yan Xie, Yulong Shen, Jinfeng Ni

**Affiliations:** a State Key Laboratory of Microbial Technology, Microbial Technology Institute, Shandong University, Qingdao 266237, China; b Institute of Health Sciences, Islamabad Campus. Khyber Medical University, Peshawar KPK, 25120 Pakistan; c CRISPR and Archaea Biology Research Centre, Microbial Technology Institute and State Key Laboratory of Microbial Technology, Shandong University, Qingdao 266237, China

**Keywords:** Fungus-growing termite, Tripartite symbiosis, Diversity, *Termitomyces*, Microbes

## Abstract

The fungus-growing termite is considered a distinct ecological niche because it involves a tripartite symbiosis between the termite host, gut microflora, and the *in vitro* fungus *Termitomyces*, which has led to the expansion of highly organized and complex societies among termite colonies. Tripartite symbiosis in fungus-growing termites may promote unique microbes with distinctive metabolic pathways that may serve as valuable resources for developing novel antimicrobial therapeutic options. Recent research on complex tripartite symbioses has revealed a plethora of previously unknown natural products that may have ecological roles in signaling, communication, or defense responses. Natural products produced by symbionts may act as crucial intermediaries between termites and their pathogens by providing direct protection through their biological activities. Herein, we review the state-of-the-art research on both microbes and natural products originated from fungus-growing termite tripartite symbiosis, highlighting the diversity of microbes and the uniqueness of natural product classes and their bioactivities. Additionally, we emphasize future research prospects on fungus-growing termite related microorganisms, with a particular focus on their potential roles in bioactive product discovery.

## Graphical abstract

**Figure:**
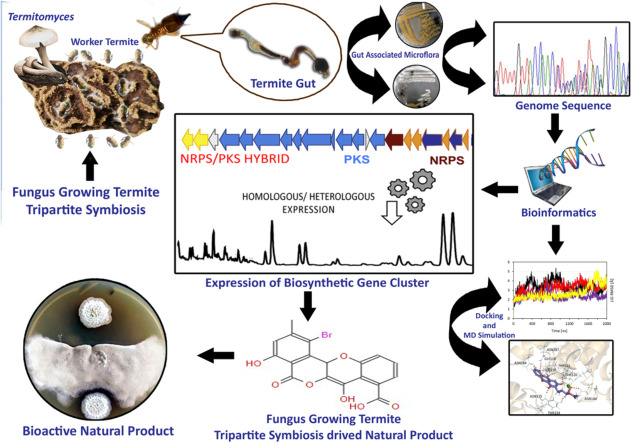

## 1 Introduction

Currently, the world faces numerous challenging environmental and health issues such as cancer, food scarcity, soil degradation, freshwater and marine pollution, climate change, and antibiotic resistance. Among these, antibiotic resistance is particularly concerning, with two million people in the United States alone contracting multi-drug resistant (MDR) infections each year, in addition to which, projections indicate that MDRs might result in 10 million deaths annually by the year 2050, if no immediate action is taken. The overuse of antibiotics is the main cause of this global health issue, as antibiotic consumption increased by 65% between 2000 and 2015 and is predicted to rise by 200% between 2015 and 2030. Given the urgency of this situation, the development of new classes of antibiotics with novel targets and methods of action has become paramount to reduce global antibiotic consumption [1], [2], [3], [4], [5]. Indeed, with drug resistance on the rise on a global scale, strengthening our capabilities for research and development of novel antibiotics is of great practical significance.

Microorganisms are the primary sources of new drugs. For more than half a century, many microbes with new antibiotics have been screened and isolated from different environments, such as soil and water. However, in recent years, the development and utilization of actinomycete resources from soil and water have reached their limits, greatly reducing the probability of discovering new drugs. Therefore, it is necessary to broaden this research field, screen new microorganisms from different sources and habitats, and discover new bioactive compounds.

Insects constitute the largest biological population in nature, accounting for over 85% of all species of living beings. Insect-associated microbes play indispensable roles in nutritional metabolism, immunity, and defense. Relevant studies have shown that insect-associated microbes can synthesize a variety of novel structural compounds [6,7], thus making it possible to find new antimicrobial drugs. Over the course of long-term evolution, some insects have developed the ability to inoculate specific fungi and cultivate them as crops. These insects are collectively referred to as fungus-growing insects, and include termites, ants, and beetles [8]. Fungus-growing termite tripartite symbiosis is perhaps the beneficial insect-microbe association that has been studied for the longest time [9]. The symbiotic relationship between *Termitomyces*, fungus-growing termites, and the gut microbiota provides valuable insights into microbial diversity and evolution, as well as potential research opportunities. This tripartite symbiosis has also resulted in the development of complex termite societies and increased the efficiency of lignocellulose decomposition, which has led to the discovery of previously unknown chemical substances with important ecological roles in signaling, communication, and defense responses. Consequently, tripartite symbiosis may foster the growth of microbes with unique metabolic mechanisms, potentially leading to the discovery of novel antimicrobial agents.

Using “fungus-growing” or “farming termites” as keywords to search the literature, we found 1047 papers (1966-2023), most of which (78.89%) were published between the year 2000 and July 27, 2023. Furthermore, 62.27% were published only over the past 12 years, indicating an increasing interest on the study of fungus-growing or farming termites. Recently, some reviews have documented natural products (NPs) originating from fungus-growing termite symbioses from a chemical-ecology perspective [10]. In this review, we searched the literature for microbes and NPs derived from tripartite symbiosis in fungus-growing termites. Subsequently, we summarized those microbes that have been identified from fungus-growing termite symbiosis using culture and non-culture methods. Our review aims to provide insight into the identity, origin, function, and activity of these microbes and new classes of NPs with potential for use in developing novel antibiotics, showing the relationship between microorganisms and bioactive products, and highlighting opportunities for future studies on termite-associated microbes in the process of unveiling and taking advantage of these NPs to develop novel therapeutic options.

## 2 Organizational ecology of fungus-growing termite tripartite symbiosis

From an anatomy/social organization combined perspective, termites are divided into lower and higher termites. Lower termites, which account for approximately 25% of all termite species, contain a large number of flagellates in their hindgut, whereas higher termites (75% of all termite species) do not develop a protozoan symbiosis within their gut [11,12]. All higher termites belong to the Termitidae family, which can be further divided into four subfamilies: *Macrotermitinae, Amitermitinae, Nasuitermitinae*, and *Termitinae*. Subfamily *Macrotermitinae*, which includes genera *Macrotermes, Odontotermes, Microtermes,* and *Ancistrotermes*, is widely distributed in tropical and subtropical regions and plays an important role in the carbon cycle on Earth [13]. Termites in this subfamily are called fungus-growing termites because they specifically cultivate Basidiomycota *Termitomyces* in their nest, thus forming a unique tripartite symbiosis system among termites and fungi *in vitro*, and intestinal bacteria, which greatly affects the life cycle of fungus-growing termites. This process has been systematically described by Li et al. [9,[14], [15], [16]]. A simple summary is as follows: older termite workers collect plant materials, chew them, and store them in their nests. In turn, young workers subsequently ingest the stored plant materials and small white cocci (fungal nudoles), which are the asexual spores of *Termitomyces*. Plant materials containing spores pass through the termite intestine and are digested by the termites and their symbiotic bacteria. Once excreted, feces form fresh combs (called fungus combs or fungus gardens). Fungal spores use combs to grow and degrade lignocellulose. Fresh combs gradually become mature combs and food for older termites. The feces ultimately excreted by older termites are stored in specialized garbage-storage areas. During heavy rain events, fruiting bodies grow from mature combs, which are edible mushrooms with high nutritional value.

## 3 Microbial communities harbored by the novel functional ecological niche, “fungus-growing termite”

### 3.1 Isolated microbes from the guts and combs of fungus-growing termites

The microbial communities that fungus-growing termites harbor in their gut and fungus comb are thought to play a role in cellulose-hemicellulose hydrolysis, biocontrol and defense, gut fermentation, synthesis of nutrients, breakdown of the fungus comb, and the beginning of the growth of the symbiotic fungi “*Termitomyces*” [17]. The bacterial gut-microflora of fungus-growing termites comprises a few dominant phyla with distinct differences among the major host groups, an issue that will be discussed in detail later. Gut microflora of fungus-growing termites, mostly harbored by bacterial lineage, particularly by the termite-specific lineages, mostly remain uncultured, although numerous research groups have worked on the isolation, identification, and characterization of microbes from the gut and associated combs to infer their possible role in tripartite symbiosis. Thus, diverse bacteria belonging to the phyla *Actinobacteria, Bacteroidetes, Firmicutes, Proteobacteria*, and *Spirochaetes*, and fungi, mainly within phylum *Basidiomycota*, have been successfully isolated (Table 1).

**Table 1:** Isolated microbes from the guts and combs of fungus-growing termites.

Fungus-growing termites	Microbial strains	Phylum	Sources	Methods	Function	References
	*Actinobacteria* (83 species)	*Actinobacteria*	Termite gut	Culturing	Immunity and protection against pathogen	[28]
	Fungal isolates (18), Ascomycotina (15), Mucorimycotina (2), Basidiomycotina (1) including *Xylaria escharoidea, X. brunneovinosa,Termitomyces* sp. *Trichoderma* etc	*Ascomycotina*	Termite gut and Fungus comb	Culturing	Symbiosis and inhibition, protect against other pathogenic microorganisms	[26]
	*Cellulomonas macrotermitis* sp. Nov	*Actinobacteria*	Termite hindgut	Culturing	Lignocellulosic degradation	[18]
*Macrotermes barneyi*	*Dysgonomonas macrotermitis* sp. Nov	*Firmicutes*	Termite hindgut	Culturing	Not Given	[97]
	*Nocardia macrotermitis*	*Actinobacteria*	Termite gut	Culturing	Not Given	[30]
	*Nocardia aurantia*	*Actinobacteria*	Termite gut	Culturing	Not Given	[30]
	*Actinomadura rubteroloni*	*Actinobacteria*	Termite gut	Culturing	The strain produced the following major phospholipids: diphosphatidylglycerol, phosphatidylethanolamine, phosphatidylinositol, phosphatidylinositol-mannoside and phosphatidylserine.	[31]
	*Actinomadura macrotermitis*	*Actinobacteria*	Termite gut	Culturing	Not Given	[31]
	*Streptomyces smaragdinus*	*Actinobacteria*	Termite gut	Culturing	Not Given	[29]
	*Actinobacteria* (97 species)	*Actinobacteria*	Termite gut (68), Termite abdomen (13), Fungus comb (16)	Culturing	Not Given	[6]
	*Actinobacteria* (67 species)	*Actinobacteria*	Termite gut	Culturing	Some bacteria can produce antibacterial substances	[6]
	*Actinomadura* sp. 5-2	*Actinobacteria*	Termite gut	Culturing	Antifungal activity	[98]
*Macrotermes natalensis*	*Trabulsiella odontotermitis*	*Proteobacteria*	Termite gut	Culturing	Possible roles in carbohydrate metabolism and aflatoxin degradation	[99]
*Macrotermes* sp., *Microtemes* sp., *Odontotermes* sp.	*Termitomyces* sp. (23 species)	*Basidiomycota*	Fungus comb and Termite gut	Culturing	Not Given	[22]
	*Sugiyamaella mastotermitis sp. nov.*	*Basidiomycota*	Termite gut	Culturing	Lignocellulosic degradation	[100]
*Mastotermes darwiniensis*	*Cellulosimicrobium* variabile sp. nov.	*Actinobacteria*	Termite hindgut	Culturing	Lignocellulosic degradation, inhibition of the growth of antagonistic bacteria	[101]
	*Klebsiella variicola*	*Proteobacteria*	Termite gut	Culturing	Nitrogen fixing activity	[19]
	*Xylaria escharoidea*	*Ascomycota*	Termite nest (Fungus comb)	Culturing	Antioxidant properties	[24]
	*Ochrobactrum* sp.	*Proteobacteria*	Fungus comb and termite gut	Culturing	Lignocellulosic degradation	[102]
	*Ɣ-Proteobacteria* (JN000928)	*Proteobacteria*	Termite gut	Culturing		[102]
	*Bacillus* sp. (JN000910-JN000913)	*Firmicutes*	Fungus comb	Culturing		[102]
	*Bacillus thuringiensis* (JN000915)	*Firmicute*	Fungus comb	Culturing		[102]
	*Bacillus* sp. (JN000920, JN000923)	*Firmicute*	Termite gut	Culturing		[102]
	*Lactococcus garviea*	*Firmicutes*	Termite gut	Culturing		[102]
	*Candida orthopsilosis*	*Ascomycota*	Fungus comb	Culturing		[102]
	*Endothia* spp.	*Ascomycota*	Fungus comb	Culturing		[102]
	*Bacillus* sp. MGB1	*Firmicutes*	Fungus comb and termite gut	Culturing	Promote the growth of abietia gallinaceus and inhibit Trichoderma harzianum.	[102]
	*Streptomyces koyangensis* BY-4	*Actinobacteria*	Termite gut	Culturing	Spectral antibacterial activity.	[68]
	*Termitomyces albuminosus*	*Basidiomycota*	Fungus comb	Culturing	Lignocellulosic degradation	[103]
	*Trabulsiella odontotermitis*	*Proteobacteria*	Termite gut	Culturing	Not Given	[104]
	*Comamonas odontotermitis*	*Proteobacteria*	Termite gut	Culturing	Not Given	[105]
*Odontotermes formaosanus*	*Xylaria acuminatilongissima, X. atrodivaricata, X. brunneovinosa, X. griseosepiacea, X. intraflava and X. ochraceostroma,X. cirrata, X. escharoidea and X. nigripes* (Nine species, the first six are newly described species, and the latter 3 are previously known species)	*Basidiomycota*	Termite nest and Fungus comb	Culturing	Not Given	[25]
	*Papiliotrema odontotermitis f.a.,* sp. *nov.*	*Basidiomycota*	Termite gut	Culturing	Lignocellulosic degradation	[100]
	*Papiliotrema odontotermitis*	*Basidiomycota*	Termite gut	Culturing	Lignocellulosic degradation	[100]
	*Spirochaeta odontotermitis*	*Spirochaetes*	Termite gut	Culturing	Not Given	[106]
*Odontotermes obesus*	*Spirochaeta odontotermitis* sp. *nov.*	*Spirochaetes*	Termite gut	Culturing	Not Given	[106]

To explore mutualistic functionality, a new bacterial species with chitinolytic and cellulolytic activities was identified in the hindgut of Macrotermes barneyi as having chitinolytic as well as cellulolytic activities [18]. Another study reported the functional aspects of the *O. formosanus* associated endophytic anaerobic isolate *Klebsiella variicola*, which showed nitrogen fixation activity [19]. Further, the novel anaerobic bacterial strain *Dysgonomonas macrotermitis* sp. was isolated from the hindgut of the fungus-growing termite *M. barney* and has been proposed to be involved in acetogenesis, based on the fermentation of a number of products [20]. Additionally, Mathew et al. (2012) studied the *O. formosanus* gut and fungus comb-associated microbiota to isolate microbes and identify their expected roles [21]. The predominant bacterial isolates belong to the genus *Bacillus* and function in lignocellulosic degradation; furthermore, these isolates inhibited the growth of the fungus *Trichoderma harzianum*, which could be a threat to symbiotic fungus *Termitomyces. In vitro* studies have suggested that *Bacillus* spp. can serve as mutualists within the microbial ecosystem of fungus-growing termites [21]. In addition, Makonde et al. (2013) reported general trends in *Termitomyces* diversity and identified 23 species of *Termitomyces* sp., which were isolated from fungal combs associated with different fungus-growing termites [22]. Another research group provided evidence of the mutualistic interaction between *O. formosanus* and the associated fungus *T. albuminosus*, focusing on their synergistic cellulase activity [23]. Some studies have focused on the isolation of *Xylaria* [24], [25], [26]. Thus, for example, Ju and Hsieh (2007) obtained six novel species, *Xylaria acuminatilongissima, X. atrodivaricata, X. brunneovinosa, X. griseosepiacea, X. intraflava*, and *X. ochraceostroma* from the fungus-growing termite-associated fungal comb of *O. formosanus* [27]. In turn, *X. escharoidea* was isolated from the termites *O. formosanus* and *M. barneyi*, showing antioxidant activity and an inhibitory role against pathogens [25,26]. To screen for potential bioactive products, numerous studies have reported work on isolation and characterization of Actinobacteria from fungus-growing termites [6,28]. Most identified species belong to the famous *Streptomyces,* which are well known to have important biosynthetic and metabolic abilities to produce antibiotics. Furthermore, some novel species belonging to the genera *Streptomyces, Nocardia*, and *Actinomadura* have been obtained and characterized from *M. barneyi* and *M. natalensis* [29], [30], [31], revealing an enormous potential of fungus-growing termites as a resource for discovering new Actinobacteria strains.

Besides bacteria and fungi, archaea are also present in the guts of lower termites, wood-feeding higher termites, and fungus-growing termites. Their role seems to be related to methanogenesis, but to the best of our knowledge, few archaea have been cultured and isolated [32,33]. Although numerous studies have categorized the bacterial and fungal groups related to fungus-growing termites, viruses linked to these exclusive ecosystems have not been investigated extensively. The only study we found in this respect was published by Kerr et al. (2018), who reported a virus of fungus-growing termite, named termite-associated circular virus 1-4, which is most comparable to associates of the family Genomoviridae and is extensively spotted in termite fungal combs in Africa. However, the possible roles of these viruses await elucidation [34].

### 3.2 Microbial diversity and community structure in the intestinal tract and fungal garden of fungus-growing termites

Fungus-growing termite guts and fungal comb-associated microbial diversification and multiplicity have been exploited mainly using 16s rRNA-gene sequences and metagenomic analytical methods (Table 2). According to 16S rRNA-sequence analysis, dominant microflora in the gut of *Macrotermes annandalei* reportedly belongs mainly to Bacteroidetes (73%) and Firmicutes (18%). Genes encoding β-Glucosidases were found mainly distributed in bacteria from phylum Bacteroidetes, suggesting that Bacteroidetes bacteria are the main members of cellulose degradation in fungus-growing termites [35]. Based on 16S rRNA sequences used to identify the core gut microbiota, Otani et al. (2014) performed gut-community analysis using diverse fungus-growing termites, including nine termite species from the following five genera: *Macrotermes, Odontotermes, Microtermes, Ancestrotermes* and *Pseudomonasanthotermes*. Their results showed that Bacteroidetes, Firmicutes, Proteobacteria, Spirochaetes, and Synergistetes were the most common members of the 26 bacterial phyla found in fungus-growing termites, and the gut microbiota structure in fungus-growing termites was similar to that of cockroaches [36].

**Table 2:** Variation in gut uncultured normal floral across fungus-growing termite's tripartite association.

Fungus-growing termites	Sample (Caste)	Microbial diversity (Dominant phylum >5%)	Method of analysis	References
*Ancistrotermes cavithorax*	Worker	Bacteroidetes, Firmicutes, Spirochaetes, Synergistetes	The 454 pyrosequencing-based analysis of 16S rRNA gene sequences	[107]
*Ancistrotermes guineensis*	Worker	Bacteroidetes, Firmicutes, Spirochaetes	The 454 pyrosequencing-based analysis of 16S rRNA gene sequences	[107]
*Macrotermes annandalei*	Worker	Bacteroidetes, Firmicutes	The 454 pyrosequencing-based analysis of 16S rRNA gene sequences	[108]
*Macrotermes falciger*	Soldier	Bacteroidetes, Firmicutes, Proteobacteria, Euryarchaeota	16S-rRNA	[109]
*Macrotermes gilvus*	Worker	Bacteroidetes, Firmicutes, Spirochaetes	T-RFLP and clonal analysis of 16S rRNA	[110]
*Macrotermes michaelseni*	Worker	Bacteroidetes, Firmicutes, Spirochaetes	The 454 pyrosequencing-based analysis of 16S rRNA gene sequences	[40]
	Soldier	Bacteroidetes, Firmicutes, Planctomycete, Proteobacteria	16S-rRNA	[109]
	Worker	Bacteroidetes, Firmicutes, Proteobacteria, Spirochaetes, Synergistetes, Planctomycete	Metagenomic analysis	[44]
	Worker	Firmicutes, Bacteroidetes	Metagenomic analysis	[111]
	Comb	Bacteroidetes, Firmicutes, Proteobacteria	16S-rRNA	[45]
	Worker	Bacteroidetes, Firmicutes	16S-rRNA	[45]
	Soldier	Bacteroidetes, Proteobacteria	Metagenomic analysis	[42]
	Worker	Bacteroidetes, Proteobacteria, Firmicutes	Metagenomic analysis	[42]
*Macrotermes natalensis*	Queen	Firmicutes	Metagenomic analysis	[42]
*Macrotermes subhyalinus*	Worker	Bacteroidetes, Firmicutes, Proteobacteria, Spirochaetes, Synergistetes, Planctomycetes, Deferribacteres	The 454 pyrosequencing-based analysis of 16S rRNA gene sequences	[107]
	Worker	Bacteroidetes, Firmicutes, Spirochaetes	16S-rRNA	[45]
	Comb	Proteobacteria, Actinomyces, Bacteroidetes	16S-rRNA	[45]
*Microtermes* sp.	Worker	Bacteroidetes, Spirochaetes, Firmicutes, Proteobacteria, Synergistetes, Planctomycetes, Actinobacteria	The 454 pyrosequencing-based analysis of 16S rRNA gene sequences	[22,40,112]
*Microtermes toumodiensis*	Worker	Bacteroidetes, Firmicutes, Spirochaetes	The 454 pyrosequencing-based analysis of 16S rRNA gene sequences	[107]
	Comb	Bacteroidetes, Firmicutes, Proteobacteria	16S-rRNA	[45]
*Odontotermes badius*	Worker	Bacteroidetes, Firmicutes, Spirochaetes	The 454 pyrosequencing-based analysis of 16S rRNA gene sequences	[107]
	Comb	Proteobacteria, Bacteroidetes	16S-rRNA	[113]
	Comb	Firmicutes, Bacteroidetes, Proteobacteria	16S-rRNA	[114]
	Worker	Family Genomoviridae (Termite associated circular virus 1 (TaCV-1) – TaCV-4)	PCR based	[114]
	Worker	Synergistetes, Firmicutes, Bacteroidetes, Proteobacteria Spirochaetes	Pyrosequencing analysis	[46]
	Comb	Firmicutes, Bacteroidetes	16S-rRNA	[46]
*Odontotermes formosanus*	Worker	Bacteriodetes, Firmicutes, Proteobacteria, Planctomyces, (Fungus Comb)	DGGE (denaturing gradient gel electrophoresis)	[102]
	Worker	Bacteroidetes, Firmicutes, Proteobacteria, Spirochaetes,	Metagenomic analysis	[44]
*Odontotermes* sp.	Worker	Bacteroidetes, Firmicutes, Spirochaetes	The 454 pyrosequencing-based analysis of 16S rRNA gene sequences	[40]
*Odontotermes species*	Worker	Bacteroidetes, Spirochaetes, Firmicutes, Proteobacteria, Synergistetes, Planctomycetes	16S-rRNA	[112]
	Worker	Bacteroidetes, Firmicutes, Proteobacteria, Spirochaetes, Synergistetes, Planctomycetes	Pyrosequencing the whole gut metagenome	[37]
	Worker	Bacteroidetes, Firmicutes, Proteobacteria	16S-rRNA	[115]
*Odontotermes yunnanensis*	Comb	Firmicutes, Proteobacteria	16S-rRNA	[115]
*Pseudacanthotermes militaris*	Worker	Bacteroidetes, Firmicutes, Proteobacteria	The 454 pyrosequencing-based analysis of 16S rRNA gene sequences	[107]
*Pseudacanthotermes minor*	Worker	Bacteroidetes, Firmicutes, Synergistetes,	The 454 pyrosequencing-based analysis of 16S rRNA gene sequences	[107]

Similarly, metagenomic sequencing analysis on the intestinal microbes of *Odontotermes yunnanensis* revealed that the dominant flora were Bacteroidetes, Firmicutes, and Spirochaetes [37]. Particularly, research has shown that Spirochaetes in the intestines of termites participate in acetic acid production, nitrogen fixation, and cellulose degradation [38,39]. Makonde et al. (2015) compared bacterial communities in the termite gut, mound, and surrounding soils using three fungus-growing termite species belonging to the genera *Macrotermes, Odontotermes* and *Microtermes*. The results of their analysis of bacterial composition and community structure in the gut were similar to those of previous reports, with Bacteroidetes (40-58%), Firmicutes (17-27%), and Spirochaetes (10-70%) being the dominant phyla. However, in fungal combs and surrounding soil, the dominant phyla were Acidobacteria (28-45%), Actinobacteria (20-40%), and Proteobacteria (18-24%) [40]. Members of Actinobacteria are very abundant in the termite mounds. As the main producers of antimicrobial compounds, Actinobacteria help prevent the contamination of fungal gardens and inhibit the growth of certain pathogens or antagonists [28,41].

Gut microbial diversity is influenced by several factors, among which, termite castes may play a determinant role. Specifically, most research on microbial communities has focused on termite workers. Recently, however, to determine microbial diversity and function with the division of labor, Poulsen et al. (2014) compared the gut microbiota of workers, soldiers, and queens of *Macrotermes natalensis* through metagenomic sequencing analysis. They found that the gut microbiota of queens was dominated by Bacillus of Phylum Firmicutes (98.7%) with no detected genera (*Alistipes, Bacteroides, or Desulfovibrio*), which was significantly different from the gut microbiota of workers and soldiers [42]. Compared to the queen, the gut microbiota of worker and soldier termites exhibited similarity, and both were dominated by the following distinctive genera: *Alistipes, Bacteroides, Desulfovibrio*, and *Clostridium*, as well as *Burkholderia* [42]. The glycoside hydrolase gene was annotated in the sequences of worker gut microbiota and the fungus *Termitomyces*. Further analysis showed that the gut microbiota of worker termites was mainly responsible for the degradation of oligosaccharide substrates. In addition, *Streptomyces* have been identified in the gut microbiota of workers and soldiers [42].

To further explore the impact of termite castes on gut microbiota, Otani et al. (2019) used amplicon sequencing to analyze gut community composition in workers and soldiers of three different termite species (*M. natalensis, Odontotermes* cf. *badius*, and *Odontotermes* sp.) as well as reproductive termites, including the queen and king from eight species of fungus-growing termites. Enzyme activities and microscopic studies were combined to infer the roles of the microbiota and explore whether the unique lifestyle of the queen and king affected the composition of gut microbiota. The study revealed relatively small differences between minor and major workers; however, significant differences between the gut microbiota of soldiers and workers was effectively explained by their function and diet. The reduced bacterial diversity in the queen and king reflects the influence of the division of labor on the level of gut symbiosis [43].

Diet is another key factor affecting the functional composition of gut microbiota. Thus, for example, Hu et al. (2019) performed comparative analyses of gut microbiomes using nine termite species belonging to five different genera under different diets, including fungus, wood, dung, humus, litter, and soil-feeding termites [44]. This study indicated a tight link between diet and gut community composition. In the fungus-growing termites, the dominant bacteria were Bacteroidetes and Firmicutes, whereas in wood-feeding termites, Spirochaetes and Firmicutes were dominant. Compared to other termites, fungus-growing termite guts contained more fungal cell wall-degrading enzyme genes, which are probably related to their fungal food source, whereas in wood-feeding termites, gut communities showed relatively more plant cell wall-degrading enzyme genes, further proving that diet is closely related to the composition of the gut microbiota of termites [44].

In the fungus-growing termite tripartite symbiosis, Otani et al. (2016) first presented a detailed comparison of bacterial communities in the gut and fungus combs [45]. Fungus-growing termites eat asexual spores of *Termitomyces*; then, when mixed plant material passes through the gut of the termite, the gut bacteria will deposit in combs. Using high-throughput sequencing of the 16S rRNA gene to determine which gut bacteria are transferred to fungal combs, the group investigated 33 combs from four fungus-growing termite species belonging to three genera: *Macrotermes, Microtermes* and *Odontotermes*. Consistent with previous reports on fungus-growing termite guts, the results indicated that there was a certain proportion of bacterial families shared by the gut and the fungal comb [36]. Moreover, there were more overlapping bacterial families between the gut and fungus combs of *Microtermes* and *Odontotermes*, indicating that the two termites had relatively few unique gut families compared with *Macrotermes*. In addition, they found that gut communities were consistent in composition within termite host species over time, whereas comb bacterial communities changed over time.

A third important factor affecting the community structure of the gut microbiota is age. Particularly, a comparative study of the hindgut microbiota and fungal combs in *O. formosanus* revealed differences between young and old workers, which may be related to their different functional roles in lignocellulose digestion [46]. Similarly, significant differences in the bacterial microbiota at the phylum and family levels were also observed between fresh and mature fungus combs. Further, the proportion of actinomycetes in workers of different ages appeared to be consistent, but their relative abundance varied between fresh and mature bacterial combs. Specifically, there were more actinomycetes in fresh combs, but their quantity was less than that in the gut microbiota of workers [46].

Overall, diet, caste, labor division, age, and gut microenvironment all affect the diversity of the termite gut microbiota, and diet is an important factor in controlling the structure of the termite gut microbiota. Similar to the gut community, the fungal comb community is also influenced by environmental factors in the surroundings, such as soil and termite mounds. Actinomycetes are not the dominant bacteria in the gut of fungus-growing termites, but they are the main bacterial species in termite mounds [40].

## 4 Natural products derived from fungus-growing termite tripartite symbiosis

The diversity of NPs produced by the fungus-growing termite tripartite association is attributed to the rich microbial community and synergistic interactions between termites, fungi, and bacteria. Further, these interactions have been shown to enhance the production of NPs and increase their bioactivity. Indeed, this tripartite association produces a wide range of NPs of diverse biosynthetic classes, including benzoquinones, alkaloids, polyketides, non-ribosomal peptides, and ribosomally synthesized peptides, which are involved in the production of compounds with various biological activities, such as antifungal, antimicrobial, and antiviral properties [47,48]. Based on our search in the scientific literature database and a comprehensive study (covering data from September 1972 to December 2020) reported by Schmidt in early 2022 [10], we constructed a small database of bioactive NPs derived from fungus-growing termite tripartite symbiosis (whole termites, *Termitomyces,* gut microbiota, and *Pseudoxylaria*) (NPs categorized Biosynthetic Class wise, Table 3, Table 4, Table 5, Table 6, Table 7, Table 8) (See Supplementary File 1 for details). This small database of bioactive NPs includes molecular formula, mass, PubChem CID, NP name, NP biosynthetic class, termite hosts, producing organisms, and identification method. Absorption, distribution, metabolism, and excretion (ADME) analyses [49] of bioactive NPs from the fungus-growing termite tripartite symbiosis were also performed. This analytical approach elucidated the intricate ways in which these compounds interact within biological systems (See Supplementary File 2 for details). ADME profiling plays a pivotal role in drug discovery by evaluating the pharmacokinetic properties of a compound and allowing researchers to predict its behavior in the human body. In what follows, we discuss NPs biosynthetic classes individually, along with their reported bioactivities and possible mechanisms of action.

**Table 3:** Comprehensive list of natural products from the Biosynthetic Class “Benzoquinones and sesquiterpenoids” discovered from fungus-growing termite a novel functional niche (Whole termites, Termitomyces, termite gut bacteria).

Molecular Formula	Mass (g/mol)	Natural Product Name	Natural Product Biosynthetic Class	PubChem CID	Termite Host	Producing Microorganism	Isolation Methods	Reported Bioactivity	References
C_15_H_24_	204.36	(E)-b-Farnesene	Terpene	5281517	*Macrotermes natalensis*	*Termitomyces* sp.	GC-MS	Not Tested	[116]
C_40_H_56_	536.90	Lycopene	Terpene	446925	*Macrotermes natalensis*	*Termitomyces heimi*	HPLC/Folin-Ciocalteu procedure	Anti-bacterial/Anti-fungal	[116]
C_30_H_48_O_3_	456.70	Betulinic acid	Terpenoid	64971	*Macrotermes natalensis*	*Termitomyces microcarpus*	HPLC-DAD-ESIMS	Anti-tumor	[117]
C_21_H_32_O_3_	332.24	Dimethylincisterol	Terpenoid	Not Given	*Macrotermes natalensis*	*Termitomyces microcarpus*	HPLC-DAD-ESIMS	Not Tested	[117]
C_28_H_46_O_3_	430.70	(22E,24R)-ergosta-7,22-diene-3β,5α,6β-triol	Terpenoid	166639900	*Macrotermes natalensis*	*Termitomyces microcarpus*	HPLC-DAD-ESIMS	Not Tested	[117]
C_28_H_44_O_3_	428.33	5α,6α-epoxy-(22E,24R)-ergosta-8(14),22-diene-3β,7α-diol	Terpenoid	Not Given	*Macrotermes natalensis*	*Termitomyces microcarpus*	HPLC-DAD-ESIMS	Not Tested	[117]
C_28_H_42_O_3_	426.31	5α,8α-epidioxy-(22E,24R)-ergosta-6,9(11),22-trien-3β-ol	Terpenoid	Not Given	*Macrotermes natalensis*	*Termitomyces microcarpus*	HPLC-DAD-ESIMS	Not Tested	[117]
C_28_H_46_O_3_	430.34	5α,8α-epidioxy-(22E,24R)-ergosta-6,22-dien-3β-ol	Terpenoid	Not Given	*Macrotermes natalensis*	*Termitomyces microcarpus*	HPLC-DAD-ESIMS	Anti-tumor	[117]
C_15_H_26_O	222.37	(+)-germacrene D-4-ol	Terpenoid	5352847	*Macrotermes natalensis*	*Termitomyces* sp. J132	GC-MS/NMR	Not Tested	[54]
C_15_H_26_O	222.37	(+)-intermedeol	Terpenoid	15560333	*Macrotermes natalensis*	*Termitomyces* sp. J132	GC-MS/NMR	Not Tested	[54]
C_15_H_24_	204.35	α-selinene	Terpenoid	10123	*Macrotermes natalensis*	*Termitomyces* sp. J132	GC-MS/NMR	Not Tested	[54]
C_15_H_24_	204.35	β-selinene	Terpenoid	519361	*Macrotermes natalensis*	*Termitomyces* sp. J132	GC-MS/NMR	Not Tested	[54]
C_15_H_24_	204.35	(−)-γ-cadinene	Terpenoid	10657	*Macrotermes natalensis*	*Termitomyces* sp. J132	GC-MS/NMR	Not Tested	[54]
C_31_H_52_O	440.70	Eburicol	Terpenoid	9803310	*Macrotermes natalensis*	*Termitomyces heimi*	GC-MS	Anti-bacterial	[118]
C_15_H_26_O_2_	238.37	Epi-Guaidiol A	Terpenoid	71719409	*Macrotermes natalensis*	*Termitomyces albuminosus*	MPLC	anti-acetylcholinesterase	[118]
C_30_H_50_O	426.70	Lanosterol	Terpenoid	246983	*Macrotermes natalensis*	*Termitomyces heimi*	GC-MS	Not Tested	[118]
C_15_H_26_O_2_	238.37	Teucdiol B	Terpenoid	Not Given	*Macrotermes natalensis*	*Termitomyces albuminosus*	MPLC	Not Tested	[118]
C_15_H_26_O_2_	238.37	Teucdiol C	Terpenoid	146682638	*Macrotermes natalensis*	*Termitomyces albuminosus*	MPLC	Not Tested	[118]
C_15_H_26_O_3_	254.36	Teucdiol D	Terpenoid	146682637	*Macrotermes natalensis*	*Termitomyces albuminosus*	MPLC	Not Tested	[118]
C_15_H_28_O_3_	256.38	Teucdiol E	Terpenoid	146682639	*Macrotermes natalensis*	*Termitomyces albuminosus*	MPLC	Not Tested	[118]
C_15_H_28_O_3_	256.38	Teucdiol F	Terpenoid	146682640	*Macrotermes natalensis*	*Termitomyces albuminosus*	MPLC	Not Tested	[118]
C_19_H_16_O_7_	356.30	Termstrin A	Terpenoid	156580653	*Odontotermes formosanus*	*Streptomyces* sp. BYF63	(HR)-ESI-MS	Anti-bacterial	[119]
C_21_H_18_O_9_	414.40	Termstrin B	Terpenoid	156580654	*Odontotermes formosanus*	*Streptomyces* sp. BYF63	HR-ESI-MS	Anti-bacterial	[119]
C_20_H_16_O_8_	384.30	Termstrin C	Terpenoid	156580655	*Odontotermes formosanus*	*Streptomyces* sp. BYF63	HR-ESI-MS	Not Tested	[119]
C_20_H_16_O_8_	384.30	Termstrin D	Terpenoid	156580656	*Odontotermes formosanus*	*Streptomyces* sp. BYF63	HR-ESI-MS	Anti-bacterial	[119]
C_15_H_26_O	222.37	1-epi-Cubenol	Terpene	519857	*Macrotermes natalensis*	*Termitomyces* sp.	GC-MS	Not Tested	[55]
C_11_H_18_	150.26	2-Methylenebornane	Terpenoid	123425	*Macrotermes natalensis*	*Termitomyces* sp.	GC-MS	Not Tested	[55]
C_11_H_20_O	168.28	2-Methylisoborneol	Terpenoid	16913	*Macrotermes natalensis*	*Termitomyces* sp.	GC-MS	Anti-bacterial	[55]
C_12_H_22_O	182.30	Geosmin	Terpenoid	29746	*Macrotermes natalensis*	*Termitomyces* sp.	GC-MS	Not Tested	[55]
C_13_H_22_O	194.31	Geranylacetone	Terpenoid	1549778	*Macrotermes natalensis*	*Termitomyces* sp.	GC-MS	Anti-bacterial	[55]
C_15_H_24_	204.35	Isobazzanene	Terpene	14830703	*Macrotermes natalensis*	*Termitomyces* sp.	GC-MS	Not Tested	[55]

**Table 4:** Comprehensive list of natural products from the Biosynthetic Class “Fatty acid/Fatty acid amide and Phenylpropanoid” discovered from fungus-growing termite a novel functional niche (Whole termites, Termitomyces, termite gut bacteria).

Molecular Formula	Mass (g/mol)	Natural Product Name	Natural Product Biosynthetic Class	PubChem CID	Termite Host	Producing Microorganism	Isolation Methods	Reported Bioactivity	References
C_26_H_39_NO_3_	413.60	Termitomycamide A	Fatty acid amide	49844192	*Macrotermes natalensis*	*Termitomyces titanicus*	HPLC	Not Tested	[120]
C_28_H_40_N_2_O_2_	436.60	Termitomycamide B	Fatty acid amide	97183939	*Macrotermes natalensis*	*Termitomyces titanicus*	HPLC	protective activity against endoplasmic reticulum stress-dependent cell death	[120]
C_28_H_44_N_2_O_3_	456.70	Termitomycamide C	Fatty acid amide	Not Given	*Macrotermes natalensis*	*Termitomyces titanicus*	HPLC	Not Tested	[120]
C_24_H_44_N_2_O_3_	408.60	Termitomycamide D	Fatty acid amide	49844330	*Macrotermes natalensis*	*Termitomyces titanicus*	HPLC	Not Tested	[120]
C_26_H_41_NO_2_	399.60	Termitomycamide E	Fatty acid amide	44222268	*Macrotermes natalensis*	*Termitomyces titanicus*	HPLC	protective activity against endoplasmic reticulum stress-dependent cell death	[120]
C_6_H_6_O_3_	126.11	Pyrogallol	Phenylpropanoid	1057	*Macrotermes natalensis*	*Termitomyces clypeatus*	HPLC	Anti-bacterial	[116]
C_9_H_16_O_4_	188.22	Azelaic acid	Fatty acid/hydrocarbon	2266	*Odontotermes formosanus*	*Termitomyces heimi*	LC-MS	Anti-bacterial/Anti-fungal	[41]
C_7_H_6_O_2_	122.12	4-hydroxybenzaldehyde, para-hydroxybenzaldehyde	Phenylpropanoid	126	*Odontotermes formosanus*	*Termitomyces heimi*	GC-MS	Anti-bacterial/Anti-fungal	[121]
C_14_H_6_O_8_	302.19	Ellagic Acid	Phenylpropanoid	5281855	*Macrotermes natalensis*	*Termitomyces robustus*	HPLC	Anti-bacterial	[59]
C_17_H_32_O	252.40	(R)-(-)-14-methyl-8-hexadecyn-1-ol	Fatty acid/hydrocarbon	10944926	*Odontotermes formosanus*	*Termitomyces* sp.	GC-MS	Not Tested	[55]
C_53_H_93_N_7_O_13_	1034.70	C15-surfactin	Lipopeptides	Not Given	*Odontotermes formosanus*	*Bacillus siamensis*	LC-MS	Anti-fungal	[17]

**Table 5:** Comprehensive list of natural products from the Biosynthetic Class “Alkaloids” discovered from fungus-growing termite a novel functional niche (Whole termites, Termitomyces, termite gut bacteria).

Molecular Formula	Mass (g/mol)	Natural Product Name	Natural Product Biosynthetic Class	PubChem CID	Termite Host	Producing Microorganism	Isolation Methods	Reported Bioactivity	References
C_10_H_13_NO_2_	179.22	2-ethyl-7-hydroxy-6,7-dihydro-5H-indolizin-3-one	Alkaloid	71665687	*Odontotermes formosanus*	*Streptomyces koyangensis* BY-4	HPLC	Anti-bacterial/Anti-fungal	[68]
C_11_H_12_N_2_O_2_	204.22	Banegasine	Alkaloid	10035803	*Macrotermes natalensis*	*Actinomadura* sp. RB29	LC-MS	Anti-bacterial	[6]
C_6_H_8_N_2_	108.14	2,3-Dimethylpyrazine	Alkaloid	22201	*Macrotermes natalensis*	*Termitomyces* sp.	GC-MS	Not Tested	[55]
C_6_H_8_N_2_	108.14	2,5-Dimethylpyrazine	Alkaloid	31252	*Macrotermes natalensis*	*Termitomyces* sp.	GC-MS	Not Tested	[55]
C_5_H_5_NOS	127.17	2-Acetylthiazole	Alkaloid	520108	*Macrotermes natalensis*	*Termitomyces* sp.	GC-MS	Not Tested	[55]
C_6_H_4_N_2_	104.11	2-Cyanopyridine	Alkaloid	7522	*Macrotermes natalensis*	*Termitomyces* sp.	GC-MS	Not Tested	[55]
C_5_H_6_N_2_	94.11	Methylpyrazine	Alkaloid	7976	*Macrotermes natalensis*	*Termitomyces* sp.	GC-MS	Anti-fungal	[55]
C_8_H_12_N_2_	136.19	Tetramethylpyrazine	Alkaloid	14296	*Macrotermes natalensis*	*Termitomyces* sp.	GC-MS	Not Tested	[55]
C_7_H_10_N_2_	122.17	Trimethylpyrazine	Alkaloid	26808	*Macrotermes natalensis*	*Termitomyces* sp.	GC-MS	Not Tested	[55]
C_18_H_23_N_5_O_6_	405.40	Roseoflavin	Alkaloid	49867612	*Odontotermes formosanus*	*Streptomyces davaonensis*	ESI-MS	Anti-bacterial	[122]
C_17_H_21_N_5_O_6_	391.14	8-methylamino-8-demethyl-D-riboflavin	Alkaloid	Not Given	*Odontotermes formosanus*	*Streptomyces davaonensis*	ESI-MS	Not Tested	[122]
C_5_H_5_NO	95.10	2-Formylpyrrole	Alkaloid	13854	*Macrotermes natalensis*	*Termitomyces* sp.	GC-MS	Cytotoxicity toward cancer cells	[123]

**Table 6:** Comprehensive list of natural products from the Biosynthetic Class “Polyketide Synthase PKS” discovered from fungus-growing termite a novel functional niche (Termite body extracts, Termitomyces, termite gut bacteria, *Pseudoxylaria*, fungal isolates).

Molecular Formula	Mass (g/mol)	Natural Product Name	Natural Product Biosynthetic Class	PubChem CID	Termite Host	Producing Microorganism	Isolation Methods	Reported Bioactivity	References
C_22_H_16_O_6_	376.40	Resistomycin	PKS	135430323	*Odontotermes formosanus*	*Streptomyces canus* BYB02	LC-MS	Anti-fungal	[130]
C_19_H_12_O_6_	336.30	Tetracenomycin D	PKS	443788	*Odontotermes formosanus*	*Streptomyces canus* BYB02	LC-MS	Not Tested	[130]
C_29_H_40_N_2_O_9_	560.60	Geldanamycin	PKS	5288382	*Macrotermes natalensis* Mn802	*Streptomyces* sp. M56	LC-MS	Anti-fungal	[131]
C_35_H_50_N_2_O_11_	674.80	Natalamycin A	PKS	101881152	*Macrotermes natalensis* Mn802	*Streptomyces* sp. M56	LC-MS	Anti-fungal	[131]
C_21_H_35_NO_2_	333.50	Tyroscherin	PKS	11371027	*Nasutitermes corniger*	*Pseudallescheria boydii*	LC-MS	Anti-bacterial	[124]
C_28_H_32_O_13_	576.50	Termisoflavone A	PKS	132526855	*Macrotermes natalensis*	*Streptomyces* sp. RB1	LC-MS/1D and 2D NMR	Not Tested	[125]
C_26_H_36_N_2_O_6_	472.60	Macrotermycin A	PKS	132821222	*Macrotermes natalensis*	*Amycolatopsis* sp. M39	LC-MS	Anti-microbial	[73]
C_26_H_36_N_2_O_6_	472.60	Macrotermycin B	PKS	132821223	*Macrotermes natalensis*	*Amycolatopsis* sp. M39	LC-MS	Not Tested	[73]
C_26_H_38_N_2_O_8_	506.60	Macrotermycin C	PKS	132821224	*Macrotermes natalensis*	*Amycolatopsis* sp. M39	LC-MS	Anti-microbial	[73]
C_26_H_38_N_2_O_8_	506.60	Macrotermycin D	PKS	139591053	*Macrotermes natalensis*	*Amycolatopsis* sp. M39	LC-MS	Not Tested	[73]
C_16_H_16_O_7_	320.29	Barceloneic acid A	PKS-derived	10358625	*Macrotermes natalensis*	*Streptomyces* sp. RB108	UHPLC-MS	farnesyl-protein transferase inhibitor	[6]
C_15_H_10_O_4_	254.24	Daidzein	PKS	5281708	*Macrotermes natalensis*	*Streptomyces* sp. RB1	LC-MS	Anti-bacterial/Anti-fungal	[124]
C_25_H_26_O_10_	486.50	Fridamycin A	PKS	75614470	*Macrotermes natalensis*	*Actinomadura* sp. RB99	LC-MS/UV	Increased glucose uptake into differentiated 3T3-L1 cells	[125]
C_17_H_9_O_7_Cl	360.70	Actinospirol A	PKS	156582987	*Macrotermes natalensis*	*Actinomadura* sp. RB31	BGC activation	Not Tested	[131]
C_18_H_11_O_7_Cl	374.70	Maduralactomycin A	PKS	156582985	*Macrotermes natalensis*	*Actinomadura* sp. RB29	1D and 2D NMR	Anti-bacterial	[131]
C_15_H_14_O_6_	290.27	Catechin	PKS	9064	*Macrotermes natalensis*	*Termitomyces robustus*	HPLC	Not Tested	[60]
C_15_H_14_O_6_	290.27	Epicatechin	PKS	72276	*Macrotermes natalensis*	*Termitomyces robustus*	HPLC	Not Tested	[60]
C_21_H_20_O_12_	464.40	Isoquercitrin	PKS	5280804	*Macrotermes natalensis*	*Termitomyces robustus*	HPLC	Anti-bacterial	[60]
C_15_H_10_O_6_	286.24	Kaempferol	PKS	5280863	*Macrotermes natalensis*	*Termitomyces robustus*	HPLC	Anti-bacterial/Anti-fungal	[60]
C_27_H_30_O_16_	610.52	Rutin	PKS	5280805	*Macrotermes natalensis*	*Termitomyces robustus*	HPLC	Anti-bacterial	[60]
C_33_H_54_O_12_	642.80	1′14-Dihydroxyisochainin	Polyenes (Macrolids) PKS	139589267	*Macrotermes barneyi*	*Streptomyces* strain HF10	LC-MS	Anti-fungal	[28]
C_35_H_58_O_12_	670.80	Pentamycin	Polyenes (Macrolids) PKS	5282200	*Macrotermes barneyi*	*Streptomyces* strain HF10	LC-MS	Anti-fungal	[28]
C_10_H_10_O_3_	178.18	4,8-dihydroxy-3,4-dihydronaphthalen-1(2H)	PKS-Ⅱ	13369486	*Macrotermes barneyi*	*Xylaria escharoidea*	ESI-MS	Anti-bacterial	[26]
C_7_H_6_O_3_	138.12	2-Methoxy-1,4-benzoquinone	PKS	76146	*Macrotermes natalensis*	*Termitomyces* sp.	GC-MS	Not Tested	[55]
C_11_H_8_O_3_	188.18	2-Methoxy-1,4-dihydroxybenzene	PKS	19833361	*Macrotermes natalensis*	*Termitomyces* sp.	GC-MS	Not Tested	[55]
C_31_H_27_O_10_	559.20	Grincamycin N	PKS	Not Given	*Odontotermes formosanus*	*Streptomyces lannensis* BYF106	LC-MS	Anti-bacterial	[127]

**Table 7:** Comprehensive list of natural products from the Biosynthetic Class “Nonribosomal Peptide Synthetases NRPS” discovered from fungus-growing termite a novel functional niche (Termitomyces, termite gut bacteria, and *Pseudoxylaria* sp.).

Molecular Formula	Mass (g/mol)	Natural Product Name	Natural Product Biosynthetic Class	PubChem CID	Termite Host	Producing Microorganism	Isolation Methods	Reported Bioactivity	References
C_14_H_14_O_3_N_2_Cl_2_	329.20	cyclo(*N*Me-L-3,5-dichlorotyrosine-Dhb)	NRPS	Not Given	*Macrotermes natalensis*	*Actinomadura* sp. RB29	LC-MS	Anti-bacterial	[6]
C_80_H_115_N_21_O_28_S_3_	1914.73	Rubrominin A	RiPPS	Not Given	*Macrotermes natalensis*	*Actinomadura* sp. RB29	LC-HRMS	Not Tested	[6]
C_83_H_120_N_22_O_29_S_3_	1985.77	Rubrominin B	RiPPS	Not Given	*Macrotermes natalensis*	*Actinomadura* sp. RB29	LC-HRMS	Not Tested	[6]
C_19_H_25_N_3_O_5_	375.40	Natalenamide A	NRPS	146684094	*Macrotermes natalensis*	*Actinomadura* sp. RB99	LC-MS/UV	Weak cytotoxicity against HepG2 liver cancer cells	[124]
C_20_H_27_N_3_O_5_	389.40	Natalenamide B	NRPS	146684095	*Macrotermes natalensis*	*Actinomadura* sp. RB99	LC-MS/UV	Weak cytotoxicity against HeLa cervical cancer cells and A549 human lung cancer cells	[124]
C_23_H_25_N_3_O_5_	423.50	Natalenamide C	NRPS	146684096	*Macrotermes natalensis*	*Actinomadura* sp. RB99	LC-MS/UV	Inhibitory effects on IBMX-mediated melanin synthesis	[124]
C_62_H_86_N_12_O_16_	1255.40	*Actinomycin* D	NRPS	457193	*Odontotermes formosanus*	*Streptomyces* sp. T33	LC-MS/NMR	Anti-bacterial/Anti-fungal	[128]
C_7_H_10_N_2_O_2_	154.17	Pyrrolo[1,2-a]pyrazine-1,4-dione, hexahydro-	NRPS	193540	*Odontotermes formosanus*	*Termitomyces heimi*	GC/MS	Anti-bacterial	[121]
C_11_H_18_N_2_O_2_	210.27	Pyrrolo[1,2-a]pyrazine-1,4-dione, hexahydro-3-(2-methylpropyl)-	NRPS	102892	*Odontotermes formosanus*	*Termitomyces heimi*	GC-MS	Anti-bacterial	[121]
C_14_H_16_N_2_O_2_	244.29	Pyrrolo[1,2-a]pyrazine-1,4-dione, hexahydro-3-(phenylmethyl)-	NRPS	99895	*Odontotermes formosanus*	*Termitomyces heimi*	GC-MS	Not Tested	[121]
C_22_H_30_N_2_O_5_	402.50	Xylacremolide A	NRPS	156582722	*Macrotermitinae*	*Pseudoxylaria* sp. X187	1D and 2D NMR	Not Tested	[85]
C_23_H_32_N_2_O_5_	416.50	Xylacremolide B	NRPS	156582723	*Macrotermitinae*	*Pseudoxylaria* sp. X187	1D and 2D NMR	Not Tested	[85]

**Table 8:** Comprehensive list of natural products from the Biosynthetic Class “PKS-NRPS hybrid” discovered from fungus-growing termite a novel functional niche (Termite body extracts, Termitomyces, termite gut bacteria, *Pseudoxylaria*, fungal isolates).

Molecular Formula	Mass (g/mol)	Natural Product Name	Natural Product Biosynthetic Class	PubChem CID	Termite Host	Producing Microorganism	Isolation Methods	Reported Bioactivity	References
C_11_H_12_O_2_	176.21	Terricollene C	Phenylpropanoid	44426063	Not Found	*Neurospora* sp.	LC-(HR)MS	Not Tested	[129]
C_23_H_37_N_3_O_6_	451.60	Microtermolides A	PKS-NRPS hybrid	60149579	*Microtermes* sp.	*Streptomyces* sp. MspM5	LC-MS	Not Tested	[80]
C_22_H_35_N_3_O_7_	453.50	Microtermolides B	PKS-NRPS hybrid	60149580	*Microtermes* sp.	*Streptomyces* sp. MspM5	LC-MS	Not Tested	[80]
C_32_H_44_N_4_O_4_	548.70	Pseudoxylallemycins A	PKS-NRPS hybrid	132934654	*Microtermes* sp.	*Pseudoxylaria* sp. X802	LC-(HR)MS	Antimicrobial activity and antiproliferative and cytotoxic properties	[130]
C_40_H_52_N_4_O_6_	684.90	Pseudoxylallemycins B	PKS-NRPS hybrid	139588340	*Microtermes* sp.	*Pseudoxylaria* sp. X802	LC-(HR)MS	Antimicrobial activity and antiproliferative and cytotoxic properties	[130]
C_36_H_48_N_4_O_5_	616.80	Pseudoxylallemycins C	PKS-NRPS hybrid	137248076	*Microtermes* sp.	*Pseudoxylaria* sp. X802	LC-(HR)MS	Antimicrobial activity and antiproliferative and cytotoxic properties	[130]
C_36_H_48_N_4_O_6_	632.80	Pseudoxylallemycins D	PKS-NRPS hybrid	137248077	*Microtermes* sp.	*Pseudoxylaria* sp. X802	LC-(HR)MS	Antimicrobial activity and antiproliferative and cytotoxic properties	[130]
C_36_H_50_N_4_O_5_	618.80	Pseudoxylallemycins E	PKS-NRPS hybrid	132934657	*Microtermes* sp.	*Pseudoxylaria* sp. X802	LC-(HR)MS/1D and 2D NMR	Not Tested	[130]
C_40_H_54_N_4_O_6_	686.90	Pseudoxylallemycins F	PKS-NRPS hybrid	137248078	*Microtermes* sp.	*Pseudoxylaria* sp. X802	LC-(HR)MS/1D and 2D NMR	Not Tested	[130]
C_39_H_65_N_9_O_14_	884.00	Dentigerumycin B	PKS-NRPS hybrid	139591057	*Macrotermes natalensis*	*Streptomyces* sp. M41	LC-MS (SI)	Not Tested	[90]
C_35_H_60_N_8_O_12_	784.90	Dentigerumycin C	PKS-NRPS hybrid	132490713	*Macrotermes natalensis*	*Streptomyces* sp. M41	LC-MS (SI)	Not Tested	[90]
C_35_H_61_N_9_O_11_	783.90	Dentigerumycin D	PKS-NRPS hybrid	132490714	*Macrotermes natalensis*	*Streptomyces* sp. M41	LC-MS (SI)	Not Tested	[90]
C_34_H_48_N_2_O_6_	580.80	Bacillaene A	PKS-NRPS hybrid	25144999	*Macrotermes natalensis*	*Bacillus* sp.	LC-MS	Anti-bacterial	[41]
C_14_H_14_O_3_	230.26	Eucalyptene A	Phenylpropanoid	10014072	Not Found	*Pseudoxylaria* sp. X808	LC-(HR)MS	Not Tested	[126]
C_35_H_46_O_5_N_4_	603.35	Pseudoxylallemycin G1-M3	PKS-NRPS hybrid	Not Given	Not Found	*Pseudoxylaria* sp. X819	UHPLC‐MS	Anti-bacterial	[126]
C_35_H_50_N_4_O_6_	622.80	Pseudoxylaramide A	PKS-NRPS hybrid	156582718	Macrotermitinae	*Pseudoxylaria* sp. X187	1D and 2D NMR	Not Tested	[85]
C_34_H_48_N_4_O_6_	608.80	Pseudoxylaramide B	PKS-NRPS hybrid	156582719	Macrotermitinae	*Pseudoxylaria* sp. X187	1D and 2D NMR	Not Tested	[85]
C_38_H_48_N_4_O_6_	656.80	Pseudoxylaramide C	PKS-NRPS hybrid	156582720	Macrotermitinae	*Pseudoxylaria* sp. X187	1D and 2D NMR	Not Tested	[85]
C_37_H_46_N_4_O_6_	642.80	Pseudoxylaramide D	PKS-NRPS hybrid	156582721	Macrotermitinae	*Pseudoxylaria* sp. X187	1D and 2D NMR	Not Tested	[85]
C_30_N_37_NO_7_	523.60	19, 20-Epoxycytochalasin Q	PKS-NRPS hybrid	44559501	Not Found	*Xylaria obovata* ADA-228	1D and 2D NMR	Anti-fungal	[131]

### 4.1 Fatty acid/fatty acid amide and benzoquinones

A plethora of NPs have been explored in the intriguing realm of fungus-growing termite tripartite symbiosis, encompassing diverse classes, such as benzoquinones, sesquiterpenoids, and fatty acid/fatty acid amides. These secondary metabolites play important roles in shaping the dynamic interactions among termites, cultivated fungi, and their mutualistic microbial partners. Specifically, benzoquinones [10] and sesquiterpenoids [50] are a class of NPs synthesized via the shikimate and mevalonate/non-mevalonate pathways, which typically contain a benzene ring with two carbonyl groups (quinone). They have been isolated from various sources, including fungi, plants, and bacteria. Specifically, antifungal and antibacterial benzoquinones are present in the whole termite body secretions of *Odontotermes, Macrotermes*, and *Microtermes* soldiers. These compounds may play important roles in the protection of termite colonies and fungal gardens from pathogens and predators. Recently, four novel selinane-type sesquiterpenoids, teucdiols C-F, have been identified in *T. albuminosus*. Specifically, these experimental results highlight the potential use of NPs as a therapeutic option for Alzheimer's disease [51]. Another study reported four anthraquinone derivatives, termstrins A-D, obtained from the fungus-growing termite-associated *Streptomyces* sp. BYF63, which have moderate antibacterial activity [52]. Recently, another group reported three terpene synthases that were functionally characterized as intermedeol synthase, cadinene synthase, and germacrene D-4-ol synthase from termite-associated *Termitomyces*. Terpene synthases play a crucial role in the discovery of NPs because they catalyze the formation of terpenes [53]. In an analysis of *M. bellicosus* soldier extracts, one research group discovered high yields of hydro-quinone and methyl hydro-quinone, both of which have antibacterial properties [54]. Consequently, the production of benzoquinones and sesquiterpenoids by both termites and associated fungi appears to play an important role in maintaining the mutualistic nature of symbiosis [55,56] (Table 3).

More than 50 NPs characterized from termite-associated *Termitomyces* are fatty acids/fatty acid amides, which play pivotal roles as secondary metabolites/NPs in their native host ecology and, in many cases, display anti-infective bioactivities [57]. Among these compounds, four novel glycolipids, namely, termitomycesphins A–D, have been reported in termite-associated *T. albuminosus*, and were shown to induce neuronal differentiation in rat PC12 cells but no antibacterial activity was tested [58]. Another research group investigated the antagonistic potential of *B. siamensis* YC-9 obtained from the nest of *O. formosanus,* which resulted in the isolation of five NPs, among which, C15-surfactin, a lipoprotein, showed significant antifungal activity [17]. Furthermore, myristic and ellagic fatty acids have been isolated from *T. heimii* and *T. robustus*, respectively, and have been shown to significantly inhibit gram-positive bacteria. Their mechanism of action involves inhibition of type I fatty acid synthase, which prevents the production of fatty acids, increasing membrane permeability and causing the formation of pores in the cell membranes [59,60]. Additionally, five new fatty acid amides, termite-mycamides A–E from *Termitomyces* have been discovered, two of which demonstrated protective effects against stress-dependent cell death [61] (Table 4).

### 4.2 Alkaloids and bioactive nitrogenous heterocycles

In the following section, we delve into alkaloids and bioactive nitrogenous heterocycles. Several studies have identified a total of 27 alkaloids and nitrogen heterocycles in fungus-growing termite tripartite associations. Among them, 17 have been reported from different species of *Termitomyces*, one from the fungus comb, and nine from the entire termite body. Of these, eight compounds have antimicrobial activity and five compounds have antifungal activity. These include, 2,3-Dimethylpyrazine, 2,5-Dimethylpyrazine, 2-Acetylthiazole, 2-Cyanopyridine, Tetramethylpyrazine and Trimethylpyrazine [62]. However, their specific functions in *Termitomyces* remain poorly understood. It is hypothesized that they serve as intra- and inter-organismic signal transducers and growth promoters within this novel ecological niche [10]. Indole-3-carboxaldehyde has been isolated from fungus comb of *M. natalensis* and has antifungal activity against nonfilamentous fungi and antibacterial activity against gram-positive bacteria [63]. Another indole-containing compound demonstrated synergistic effects in terms of antifungal activity against *Candida albicans* when combined with polygoides [64]. To date, scientific studies have been predominantly restricted to *Streptomyces, Amycolatopsis*, and *Actinomadura*, which belong to phylum Actinobacteria [45]. Here, we highlight the alkaloids identified from fungus-growing termite-associated bacterial symbionts that exhibit considerable antimicrobial efficacy. Recently, an Alkaloid “Banegasine” from termite associated Bacterial symbiont *Actinomadura* sp. RB29 with moderate antibacterial activity towards Gram-positive bacteria was reported [6]. Banegasine also elicits a synergistic response with pyrrolnitrin, which enhances its antibacterial and antifungal activity [65]. Similarly, two NPs, Roseoflavin and 8-methylamino-8-demethyl-d-riboflavin, identified using the conventional method from *O. formosanus* body-associated *S. davaonensis*, were reported as promising therapeutic options against Methicillin-resistant *Staphylococcus aureus* [66]. Additionally, Guo et al. (2017) used an HR-MS-based dereplication approach and feeding-based experimental strategy and identified six novel alkaloids (rubterolones A–F) from *M. natalensis* gut associated-*Actinomadura* sp. 5-2. This approach represents significant progress in the process of recognizing known compounds and offers the opportunity to delve deeper into the uncharted territory of novel molecules within these complex systems. This study indicated that gut microbes can present a diverse and prolific source of NPs [67]. Similarly, another study reported a new alkaloid (2-Ethyl-7-hydroxy-6,7-dihydro5H-indolizin-3-one) isolated from *O. formosanus* associated *S. koyangensis* BY-4, with weak antimicrobial activity [68]. Consequently, alkaloids are thought to protect termites and their fungal gardens from harmful microbes and insects, thus contributing to the stability of tripartite symbiosis [69] (Table 5).

### 4.3 Polyketides and non-ribosomally synthesized peptides

**Fig. 1:**
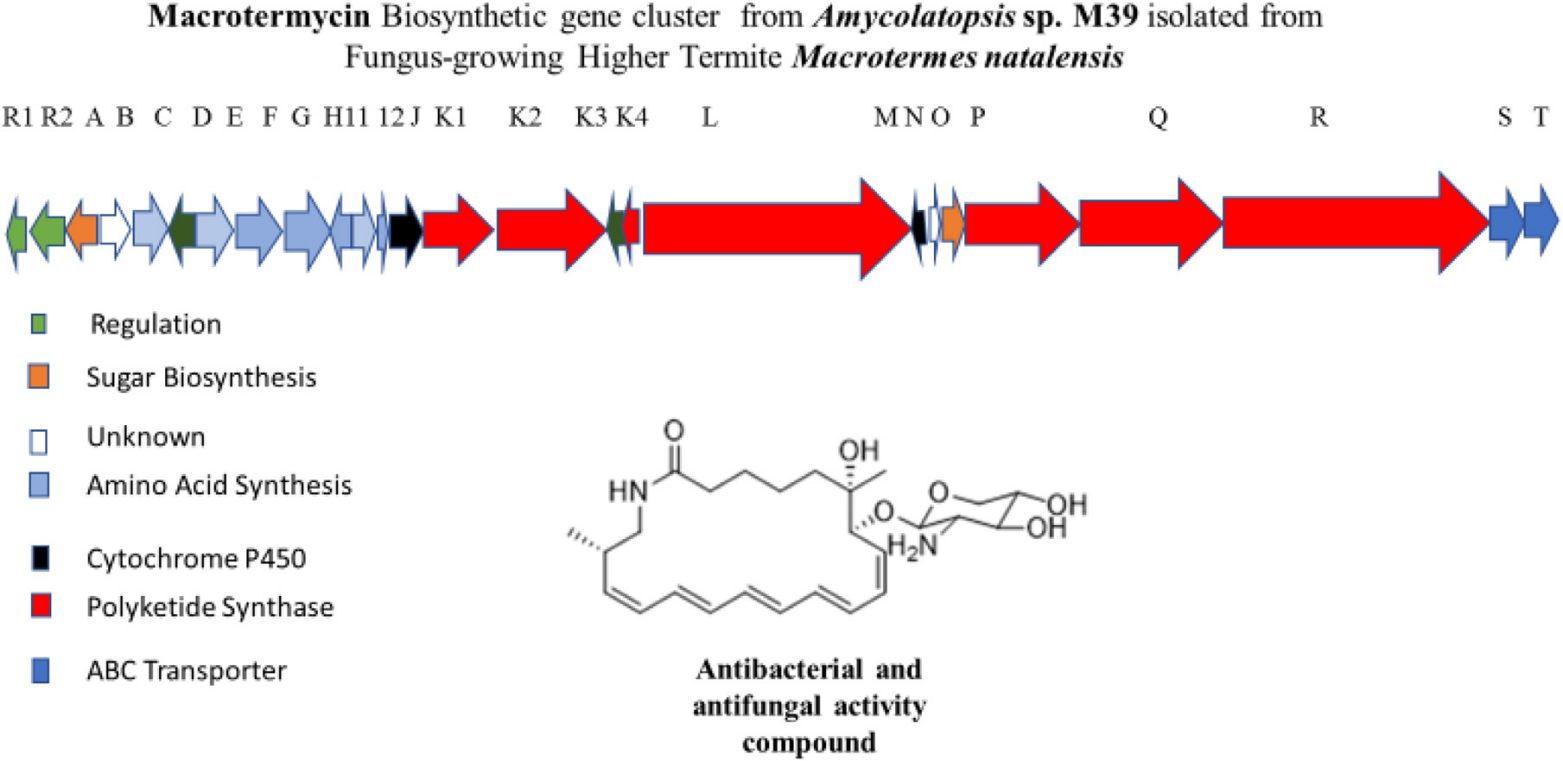
Gene map of the Macrotermycin BGC isolated from the Macrotermes natalensis gut-associated Amycolatopsis sp. Each arrow shows the direction transcription of ORF (Modified according to Ref. 76).

This section focuses on the synergistic interactions among polyketides (PKS), non-ribosomally synthesized peptides (NRPSs), and PKS-NRPS hybrids. Polyketides, also termed macrolides, include erythromycin and rapamycin and are a large class of structurally diverse biosynthetic NPs produced by numerous microorganisms that frequently demonstrate a diversified biological potential [70]. They are produced by the condensation of simple acetate and propionate building blocks via the polyketide synthase pathway, followed by further modifications, including glycosylation, methylation, and oxidation [71]. Additionally, amino benzoyl rhamnopyranoside from the cuticle of *M. natalensis*-associated *Streptomyces* sp. RB1 has been identified. This chemical investigation provides a renoprotective outcome of ABR, which may encourage the development of novel medicinal NPs to prevent cancer therapy-induced nephrotoxicity [72]. A recent bioassay-based metabolomics study aimed to characterize four novel glycosylated Macrotermycins A−D from a fungus-growing termite gut associated *Amycolatopsis* sp. M39. Concomitantly, a potential macromycin biosynthetic gene cluster sharing homology with the vicenistatin gene cluster was identified. This encompasses the biosynthesis of 3-amino-2-methylpropionate starter units. Notably, a single module lacks the dehydratase domain, terminating with a ketoreductase classified as an ‘A-type’ reductase. However, its precise biosynthetic pathway remains unclear and is currently under investigation. Macrotermycins A and C showed antibacterial activity against *S. aureus*, and antifungal activity against pathogenic fungi of the termite fungal comb (Fig. 1) [73].

The Fungus-fungus interaction-guided assays and HR-MS guided dereplication approach adopted by Guo et al. (2016) yielded six novels cyclic tetra peptides named pseudo-xylallemycins A−F, which were isolated from termite associated *Pseudoxylaria* sp. X802. These NPs showed antimicrobial activity against *P. aeruginosa* and anticancer activity against K-562 and human umbilical vein endothelial cell lines [74]. In turn, natalamycin A was identified in *Streptomyces* strain M56, which is associated with the fungal comb of *M. natalensis* [75]. Additionally, two key antifungal compounds, Resistomycin and Tetracenomycin D, were isolated and purified from termite-associated *S. canus* BYB02 using column chromatography. Resistomycin has been identified as an antifungal agent [76]. Another study reported two compounds, Microtermolides A and B, isolated from *Streptomyces* sp. associated with the termite *Microtermes* sp. [77]. Furthermore, a literature search found that microtermolide A is functionally associated with the antibiotic vinylamycin [78] (Table 6).

Non-ribosomally synthesized peptides, NRPSs, are large modular enzymes responsible for the biosynthesis of a diverse array of NPs, including antibiotics, siderophores, and immunosuppressants. NRPS-derived biomolecules are often functionally improved by post-translational modifications [79]. The study identified and characterized *Actinobacteria* from the whole gut, exoskeleton, and fungal comb of *M. natalensis*. Further analysis yielded two novel tetracyclic lanthipeptides named Rubrominins A and B [6]. Actinomycin D, a chromopeptide antibiotic, is a well-known NP isolated from *Streptomyces* and associated with fungus-growing termites. Actinomycin D is a bicyclic chromopeptide lactone composed of two non-ribosomal cyclic pentapeptide lactones and chromophoric phenoxazinone dicarboxylic acid. It appears to have a considerable impact on several ecologically significant fungi, including *Pseudoxylaria*, as well as broad-spectrum antibacterial activity [80]. This impact might be attributed to the mechanism of action of Actinomycin D, which includes the suppression of DNA-dependent RNA synthesis and presumably targeting the plasma membrane of fungi through membrane splitting mechanisms [81]. The chemical treasures of a fungal stowaway were recently studied and the targeted isolation and characterization of two cyclic NRPS-derived NPs (Xylacremolide A and B) and four linear NRPS (Pseudoxylaramide A-D) from the termite-associated *Pseudoxylaria* sp. X187 were reported. Moderate antimicrobial activity was observed in these cultures. The isolated compounds were not tested for any bioactivity [82]. Lastly, three new cyclic tripeptides, known as natalenamide A-C were identified as a result of research aimed at identifying novel pharmacologically relevant NPs. These NPs were isolated from *Actinomadura* sp. RB99 using an LC/MS/UV-based dereplication technique, and are most likely of NRPS origin. The cytotoxicity of natalenamides A and B against cancer cell lines was minimal. Additionally, melanin formation by 3-isobutyl-1-methylxanthine is markedly reduced by Natalenamide C [83] (Table 7).

**Fig. 2:**
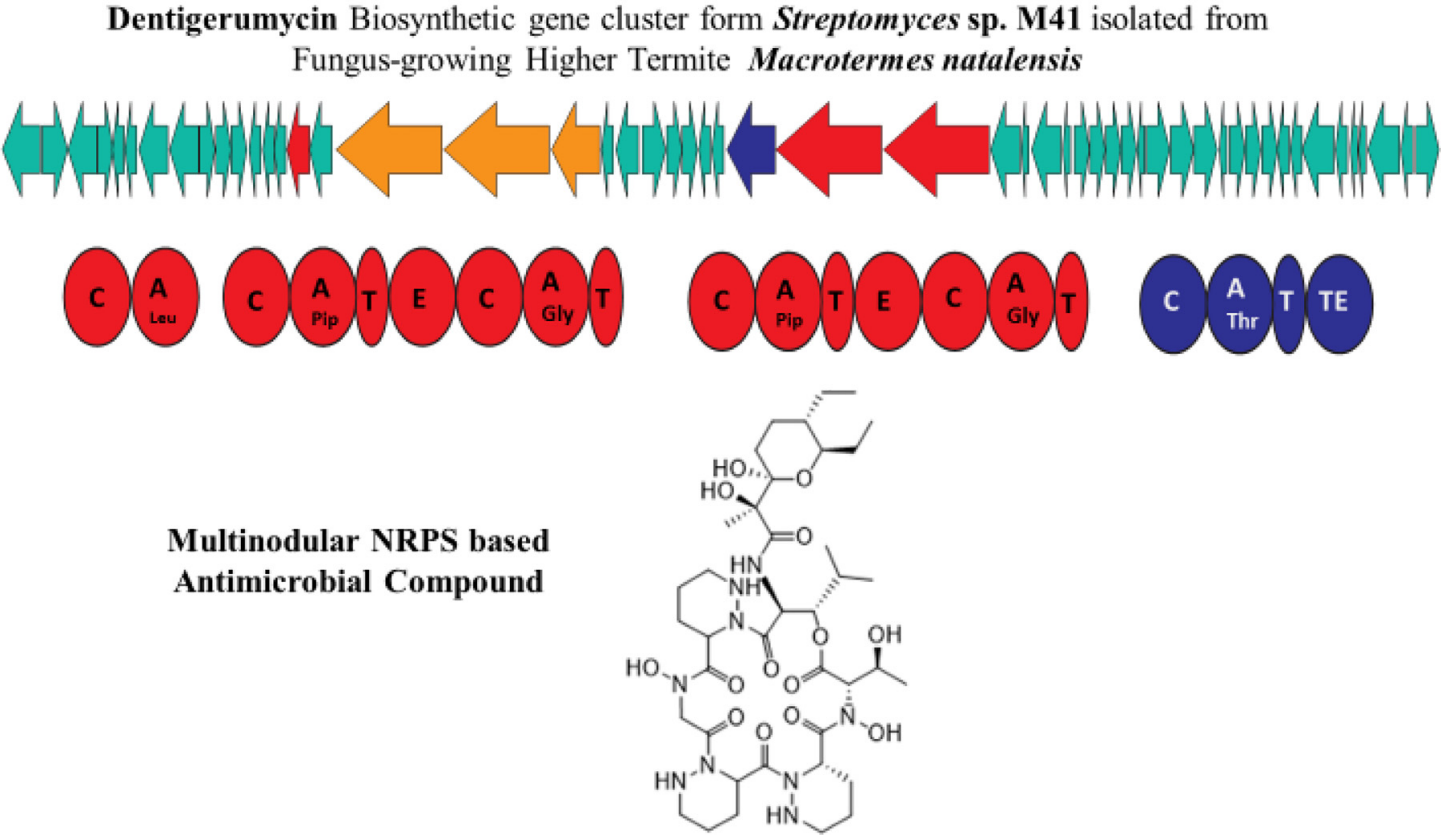
Gene map of the Dentigerumycin BGC isolated from Macrotermes natalensis gut-associated Streptomyces sp. Multinodular Non-Ribosomal Polyketide Synthases (NRPS) producing Dentigerumycin are emphasized in red and blue colors (Modified according to Ref. 90).

Polyketides-NRPS hybrids are a class of NPs produced through hybridization of polyketide synthase (PKS) and non-ribosomal peptide synthetase (NRPS) biosynthetic pathways. These hybrid NPs have been isolated from a wide range of microorganisms, including bacteria, fungi, and actinomycetes, and display diverse biological activities [79,84]. In 2013, *M. natalensis* associated bacterial symbiont *Bacillus* sp. was shown to produce Bacillaene A, a polyene polyketide, which exhibits strong broad spectrum anti-bacterial and anti-fungal activity against fungal garden antagonists [85]. Bacillaene A is known to impede the production of proteins in bacteria, possibly by preventing peptide elongation during translation. However, it is unclear how it inhibits fungal growth [86]. In a subsequent study, three novel dentigerumycin hybrid analogs (A-C) were produced by *M. natalensis* gut-associated *Streptomyces* sp. M41 [87]. Dentigerumycin B-C were tested against various Gram-positive and Gram-negative bacteria, yeast, and fungi, and showed no detectable activity [87] (Fig. 2) (Table 8). Dentigerumycin also shares structural similarities with the mycopathogen-specific antifungal dentigerumycin produced by *Pseudonocardia* [88].

In summary, the discovery of diverse NPs in the tripartite symbiosis of fungus-growing termites has several functional implications. Firstly, they may serve as a lead for the development of new drugs or agrochemicals with improved efficacy and fewer side effects. Secondly, this review provides insights into the ecological and evolutionary roles of these compounds in symbiotic relationships. Furthermore, they may provide clues about the coevolutionary interactions between a host and its symbionts, and shed light on the mechanisms underlying the maintenance of symbiotic relationships.

### 4.4 ADME profile analysis of NPs derived from fungus growing termite tripartite symbiosis

The preceding sections of our review focused on the fascinating diversity of natural products derived from the fungus-growing termite tripartite symbiosis, with an emphasis on their discovery and ecological roles. However, it is crucial to recognize that the relevance of these natural products extends beyond discovery. To bridge the gap between these intriguing discoveries and their practical applications, it is imperative to assess their pharmacological potential and safety. Therefore, we now shift our attention to the critical aspects of their potential applications.

To fully understand the practical utility of these compounds, it is imperative to scrutinize their absorption, distribution, metabolism, and excretion profiles. However, a significant bottleneck in the later stages of lead discovery is the analysis of the absorption, distribution, metabolism, excretion (ADME), and overt toxicity properties of drug candidates [89]. A pivotal rule in this domain is Lipinski's rule, also known as the Rule of Five, which correlates chemical structure with biological activity. ADME/toxicity predictions from 2D and 3D chemical structures incorporate drug-likeness modules based on this rule [90,91], which is paramount for the stepwise enhancement of pharmacologically active Pb compounds with increased activity, selectivity, and drug-like characteristics. Indeed, toxicity profoundly influences drug design and development. On the other hand, advanced computational methods play a crucial role in analyzing the physicochemical attributes of drug-like substances, particularly in elucidating likely compound mechanisms of action [92]. Moreover, this study underscores the value of sequential *in silico* methods such as ADMET analysis as essential tools for navigating chemical spaces and selecting optimal ligand structures for experimental tests, thereby minimizing testing costs. The molecular descriptors of NPs from termite symbiosis were evaluated using Lipinski's rule. Remarkably, the selected ligands fell within the molecular weight range of 290–375 (< 500) kDa, which is an essential aspect of therapeutic drug action. The ligand characteristics were also aligned with Lipinski's limits for hydrogen bond acceptors, donors, and topological polar surface areas. The latter, a strong indicator of drug molecule bioavailability, was well below the 160 Å limit. Notably, compounds 1–6 exhibited rotatable bonds, enhancing their flexibility for efficient interactions with the binding pockets [93]. These findings emphasize the significance of ADME profile analysis guided by Lipinski's rule in facilitating the selection of viable drug candidates from diverse NPs produced by fungus-growing termite symbiotic systems (See Supplementary File 2 for Details).

## 5 Future prospects and recommendations

**Exploration of untapped microbial diversity**: The study of non-culture microbes within termite guts remains an area of great interest. Future research should focus on developing innovative techniques such as single-cell genomics [94] and metagenomic binning [95] to uncover the genomic and functional diversity of these elusive microorganisms. Understanding their roles in the tripartite symbiosis and their contribution to NPs biosynthesis may lead to the discovery of novel bioactive compounds with potentially valuable applications.

**Fig. 3:**
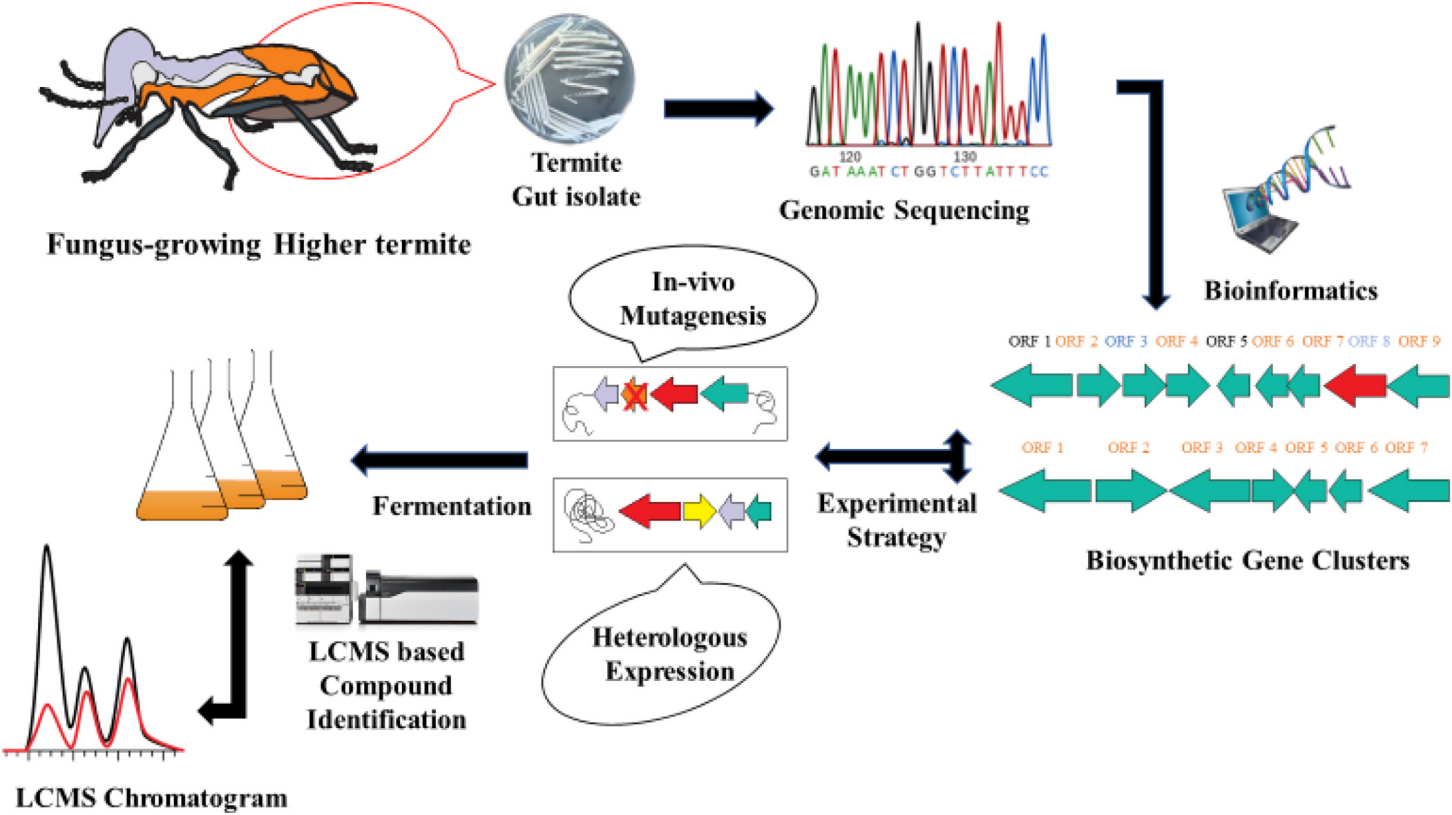
Overall genome mining strategy for novel natural product discovery from the fungus-growing termite functional ecological niche (Termite gut, Termitomyces, fungus comb, worker termite, and the whole termite-associated microflora).

**Integration of advanced omics technologies:** Embracing cutting-edge omics technologies, including metagenomics, transcriptomics, and metabolomics, will provide a more comprehensive understanding of the interplay between termite hosts, gut microbes, and *Termitomyces* fungi. Integrating these omics datasets with advanced bioinformatics tools and computational approaches will facilitate the identification of potential biosynthetic gene clusters, metabolic pathways, and regulatory elements associated with the production of natural products. Genome mining and heterologous expression are essential for unlocking bioactive natural products concealed within the intricate ecosystem consisting of a fungus-growing termite tripartite symbiosis. This approach has enabled the discovery of cryptic biosynthetic pathways and the production of valuable compounds that otherwise might have remained inaccessible. The discovery of ExoCET recombineering facilitated much to bring in the discovery of untapped diving troves for functional characterization and discovery of novel microbial NPs [96]. Heterologous expression of cryptic biosynthetic gene clusters (BCGs) for novel secondary metabolites is worthwhile for both strains, fermentation, and *in vivo* molecular manipulation of native producer strains using advanced techniques, such as the Red ET/ExoCET-based direct cloning approach (Fig. 3).

**Biotechnological applications and drug discovery:** The NPs derived from fungus-growing termites have significant potential for biotechnological and pharmaceutical applications. Future studies should focus on exploring the therapeutic potential of these compounds and their antimicrobial, antiviral, and anticancer properties. Furthermore, novel biotechnological methods, such as metabolic engineering and synthetic biology, can be utilized to boost the synthesis of bioactive compounds and facilitate sustainable efforts in bioprospecting.

**Conservation and management:** Given the ecological sensitivity of fungus-growing termite symbiosis, conservation efforts should consider preserving their unique habitats and ecosystems. Studying the impact of environmental change and human activities on this ecological niche can help formulate conservation strategies to protect this valuable source of NPs and the biodiversity they support.

**Collaborative research initiatives:** The complex nature of tripartite symbiosis in fungus-growing termites calls for interdisciplinary collaboration among researchers from diverse fields including, microbiology, ecology, chemistry, and biotechnology. Interdisciplinary collaborative efforts will foster a holistic approach to unravel the potential of this ecological niche and accelerate discoveries in natural product research.

## CRediT authorship contribution statement

**Muhammad Shoaib:** Writing – original draft, Validation, Resources, Methodology, Data curation. **Ruining Bai:** Writing – original draft, Validation, Resources, Methodology, Data curation. **Shuai Li:** Writing – original draft, Methodology, Formal analysis, Data curation. **Yan Xie:** Visualization, Validation, Data curation. **Yulong Shen:** Writing – review & editing, Visualization, Supervision, Project administration, Funding acquisition. **Jinfeng Ni:** Writing – review & editing, Supervision, Project administration, Funding acquisition, Conceptualization.

## Declaration of Competing Interest

The authors declare that they have no competing financial interests or personal relationships that may have influenced the work reported in this study.

## Appendix Supplementary materials

Image, application 1

Image, application 2
